# Three-dimensional real time imaging of amyloid β aggregation on living cells

**DOI:** 10.1038/s41598-020-66129-z

**Published:** 2020-06-16

**Authors:** Masahiro Kuragano, Ryota Yamashita, Yusaku Chikai, Ryota Kitamura, Kiyotaka Tokuraku

**Affiliations:** Graduate School of Engineering, Muroran Institute of Technology, 27-1 Mizumoto, Muroran, 050-8585 Japan

**Keywords:** Imaging, Cytoskeleton, Alzheimer's disease

## Abstract

Alzheimer’s disease (AD) is a progressive disorder of the brain that gradually decreases thinking, memory, and language abilities. The aggregation process of amyloid β (Aβ) is a key step in the expression of its neurocytotoxicity and development of AD because Aβ aggregation and accumulation around neuronal cells induces cell death. However, the molecular mechanism underlying the neurocytotoxicity and cell death by Aβ aggregation has not been clearly elucidated. In this study, we successfully visualized real-time process of Aβ_42_ aggregation around living cells by applying our established QD imaging method. 3D observations using confocal laser microscopy revealed that Aβ_42_ preferentially started to aggregate at the region where membrane protrusions frequently formed. Furthermore, we found that inhibition of actin polymerization using cytochalasin D reduced aggregation of Aβ_42_ on the cell surface. These results indicate that actin polymerization-dependent cell motility is responsible for the promotion of Aβ_42_ aggregation at the cell periphery. 3D observation also revealed that the aggregates around the cell remained in that location even if cell death occurred, implying that amyloid plaques found in the AD brain grew from the debris of dead cells that accumulated Aβ_42_ aggregates.

## Introduction

Alzheimer’s disease (AD) is a neurodegenerative disease and progressive disorder of the brain that gradually decreases thinking, memory, and language abilities^1^. The amyloid cascade hypothesis suggests that abnormal aggregation and accumulation of amyloid β (Aβ) causes neurodegeneration in the aged brain^2^. Aβ consists of 39–43 amino acid residues and is derived from amyloid precursor protein that is cleaved by β- and γ-secretase^3^. These short peptides start to aggregate and exhibit neurocytotoxicity^4^.

To appreciate the pathological feature of AD, it is important to trace the dynamic behavior of Aβ aggregation to neurons because the aggregation of Aβ is a key step in the development of AD. Several studies on the polymerization and fibrillation of Aβ have been performed using transmission electron microscopy^5–8^. Although high resolution image analysis of Aβ_42_ aggregation is possible, it is necessary to dry the sample for observation. Therefore, observation of Aβ aggregation in a physiological state cannot be performed. Recently, Querol-Vilaseca *et al*. succeeded in the three-dimensional (3D) observation of Aβ present in amyloid plaques of AD patients using stimulated emission depletion microscopy, one type of super resolution microscope^9^. Furthermore, five categories of processes in the aggregation of cellular Aβ were reported using fluorescence-lifetime imaging and 3D structural illumination microscopy^10^. Thus, although the *in situ* analysis of Aβ aggregation is increasing annually, it is difficult to perform the spatiotemporal high resolution analysis of Aβ dynamics under physiological conditions. In particular, the molecular mechanism of interaction between Aβ aggregates and the cell surface has not been clearly elucidated.

It is well known that the interaction between membrane lipids and Aβ is responsible for the modulation of Aβ fibrillation and the expression of neurocytotoxicity^11–13^. The aggregation of Aβ on the cell membrane is an important step in the formation of diffuse plaques^14^. Aβ preferentially accumulated in gangliosides and cholesterol domains of the cell membrane of PC12 cells, and these aggregates exhibited cytotoxicity^15^. Recently, using scanning electron transmission microscopy and an electron tomogram, Han *et al*. reported a 3D interaction between fibrils and the membrane of cells and that fibrils disrupted the membrane and caused leakage of lysosomes^16^. Thus, it has been shown that the cell membrane and its components can function as scaffolds for Aβ aggregation. Moreover, cell membrane morphology and the size of lipid vesicles are also important factors that modulate Aβ aggregation. Sugiura *et al*. reported that Aβ aggregation was enhanced on lipid vesicles displaying high curvature^17^. Small liposomes promoted the formation of Aβ fibrils, whereas huge liposomes inhibited the growth of Aβ fibrils, suggesting that Aβ fibrillation is dependent on liposome size^18^. Diverse morphological changes in the cell membrane are caused by cell motile processes such as cell division and migration, which are physiologically important phenomena. One cytoskeleton protein, actin, plays a particularly important and indispensable role in regulating the morphology of eukaryotic cells. Actin is especially necessary for the formation of filopodia and lamellipodia in motile cells^19^. Actin dynamics, including modulation of its assembly and disassembly, is essential for synaptic plasticity involved with memory skills^20^. Particularly, dynamics of the spine are regulated by the actin cytoskeleton^21^. Small GTPase proteins have important roles in the morphological regulation of neuron cells^20^. Rac1 activity is required for the maintenance of spine shape^22,23^. On the other hand, activation of RhoA exhibits inhibitory function in formation of spines. Overexpression of constitutively active RhoA in hippocampal neurons of mice caused the spine to retract and decrease^24^. Thus, several studies have indicated the importance of the function of actin cytoskeleton in homeostasis of neural tissue. Indeed, F-actin disassembly of the dendritic spine was caused in neuron cells of AD model mice^25^. Aβ affected the architecture of actin and the tubulin network in AD pathology^26^. However, it is obscure whether changes in cell morphology derived from cell motility influence the polymerization and fibrillization of Aβ that exists in culture medium or on the cell membrane. Furthermore, it is unknown whether morphological changes of the cellular membrane, which occur after the formation of actin-dependent protrusions, affect Aβ aggregation in the environment close to the cell surface. This implies that the direct interaction of cell membrane dynamics through actin assembly and Aβ aggregation is also unclear.

Previously, we reported a real-time imaging method of Aβ_42_ aggregation using quantum dot (QD) nanoprobes and developed a microliter-scale high-throughput screening (MSHTS) system for Aβ_42_ aggregation inhibitors that applied this imaging method^27–30^. QD, one of the fluorescent nanoparticle, is a cluster of semiconductor materials composed of hundreds to thousands of selenium and cadmium, and emits fluorescence of various wavelengths according to the particle diameter. Its size is about 10–20 nm, which is almost the same size as the protein. It is known that the advantages of QD are that excitation with a single wavelength light source is possible, and that it is very bright and hardly discolors. Since the extremely high photostability of such QD enables long-term fluorescence observation, it is most suitable for continuous observation of Aβ_42_ aggregation *in vitro*, which progresses on the order of several hours to several days. In MSHTS system, we utilized QD-conjugated Aβ_40_, which had a binding ratio (Aβ_40_/QD) of 6. We synthesized QDAβ by chemically cross-linking QD with NH_2_ group attached to the surface and Aβ_40_ with N-terminal cysteine, an amino acid having a thiol group in the side chain. By mixing QDAβ with unlabeled Aβ_42_ at a ratio of 1: 1000, we succeeded in visualizing Aβ_42_ aggregates formed by incubation at 37 °C. These QDAβ were incorporated into Aβ_42_ fibrils with a similar efficiency as unlabeled Aβ_42_. One advantage of this method is that it allows observation of the dynamics of Aβ_42_ aggregation under physiological conditions, which has been difficult until now.

In this study, we succeeded in visualizing the process of Aβ_42_ aggregation with living cells by applying the QD imaging method in real time. In other words, 2D fluorescence microscopy confirmed Aβ_42_ aggregation imaging method by QDAβ are utilized in culture medium. Further, 3D real-time observations using confocal laser microscopy revealed that Aβ_42_ preferentially started to aggregate on the dynamic cell surface where membrane protrusions frequently formed.

## Results

### Observed Aβ_42_ aggregation around PC12 cells

**Figure 1:**
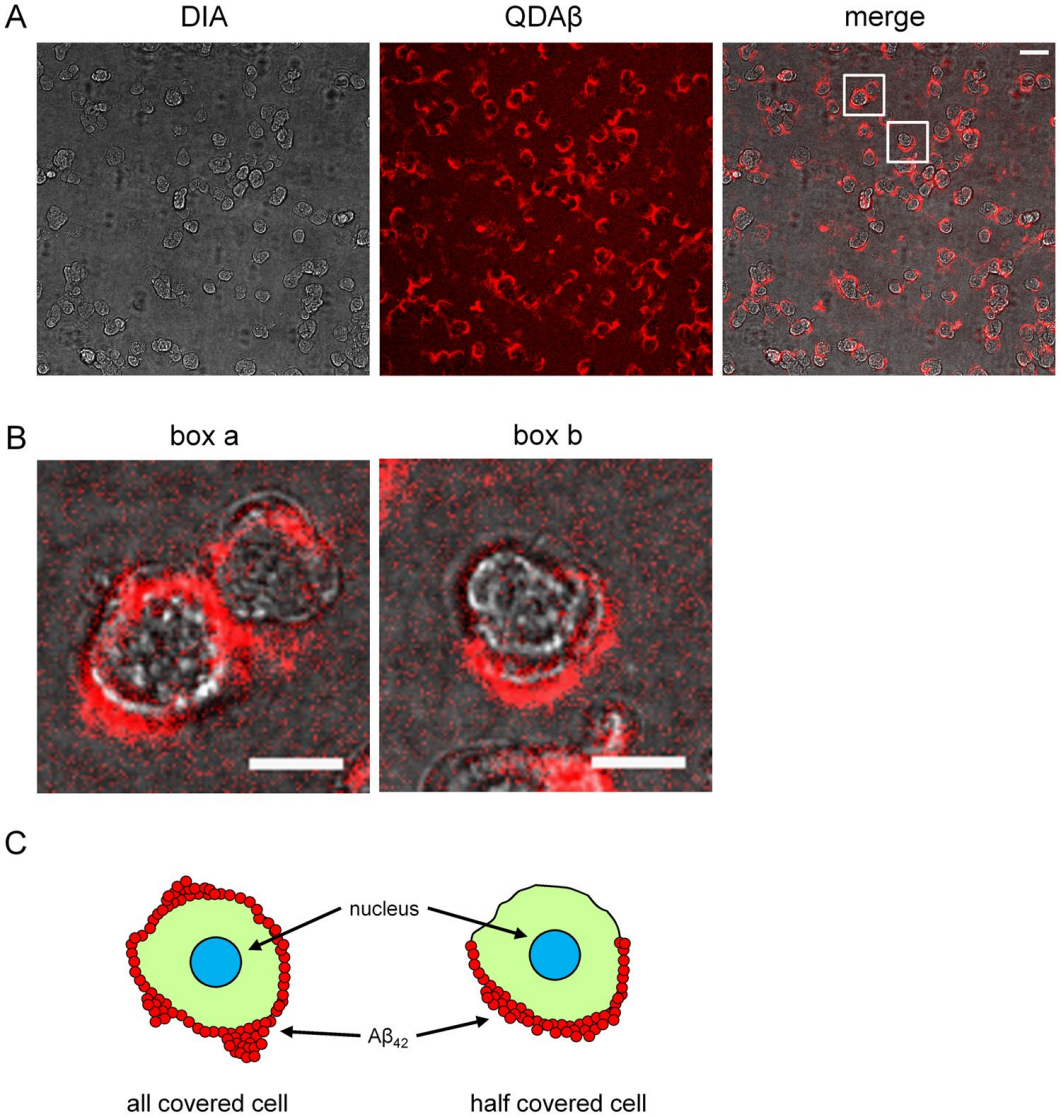
Localization and dynamics of Aβ_42_ aggregation with PC12 cells. (**A)** PC12 cells were co-incubated with 20 μM Aβ_42_ and 30 nM QDAβ, and observed by a conventional inverted microscope (DIA) and a confocal microscope (QDAβ). Bar = 50 μm. **(B**) Magnified images of boxes a and b in panel A. Bar = 20 μm. Cells were covered by Aβ_42_ aggregation on all (box a) or half (box b) of the cell periphery. (**C**) Schematic model of the Aβ_42_ aggregation with PC12 cells.

**Figure 2:**
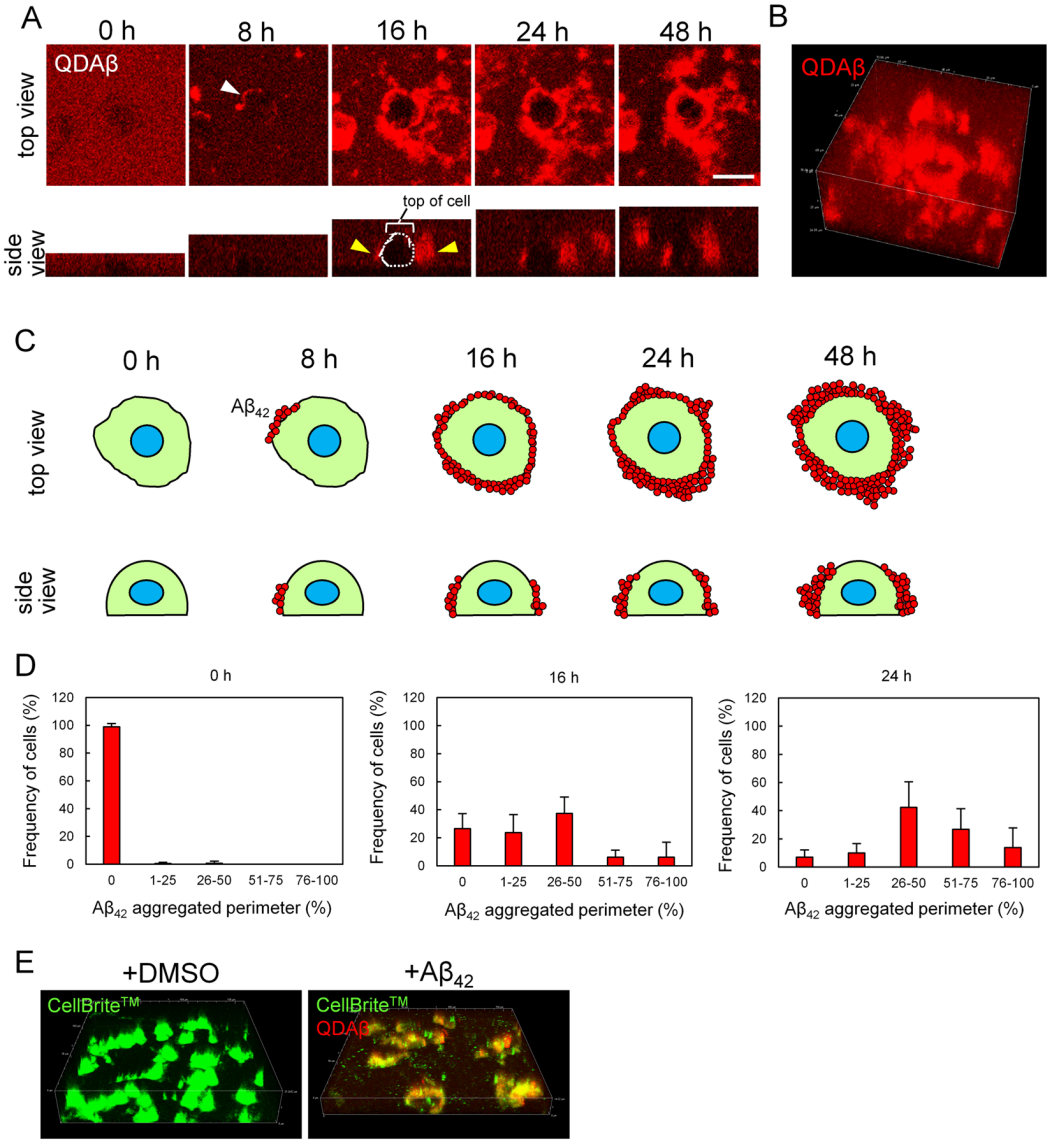
3D dynamics of Aβ_42_ aggregation with PC12 cells. (**A**) PC12 cells were co-incubated with 20 μM Aβ_42_ and 30 nM QDAβ, and then imaged by confocal microscopy with three-dimensional real-time imaging. White arrowhead indicates early aggregation at 8 h. Approximate cell morphology is shown as dotted lines at 16 h in the side view image. Yellow arrowheads indicate the accumulation of Aβ_42_ aggregates on the cell side. Note that Aβ_42_ aggregation did not localize on top of the cell. Bar = 20 μm. (**B**) 3D reconstruction of 48 h time point in Panel (**A**). (**C**) Schematic model of the Aβ_42_ aggregation around PC12 cells. Top and side view were illustrated. (**D**) Histogram of Aβ_42_ aggregated perimeter (%) after 0, 16, and 24 h incubation. The percentage of Aβ_42_-aggregated perimeter was calculated as the ratio of Aβ_42_-aggregated perimeter to the entire perimeter. n = 470, 268, 235 cells in 0, 16, 24 h incubation, respectively. Error bars represent ±SDs of the mean values of six areas in the well. Note that PC12 cells display time-dependent increase of Aβ_42_ aggregation. (**E**) 3D reconstruction of PC12 co-incubated with 30 μM Aβ_42_ and 30 nM QDAβ. Cells stained with CellBrite green (green). Images were captured by a confocal microscope. Note that Aβ_42_ aggregates (red) colocalized on the cell membrane.

We initially examined whether Aβ_42_ aggregation was observed in culture medium. Rat adrenal pheochromocytoma PC12 cells, which were differentiated by 4.5 ng/ml nerve growth factor (NGF), were then incubated with 20 μM Aβ_42_ and 30 nM QDAβ for 24 h. After incubation, we observed Aβ_42_ aggregates using a conventional fluorescence microscope (Supplementary Fig. S1) and confocal laser microscope (Fig. 1A). These observations showed that Aβ_42_ aggregates were particularly concentrated around cells. We found cells in which the entire cell periphery was covered by Aβ_42_ aggregates and other cells in which only a part of the cell was covered (Fig. 1B,C). We confirmed that NG108–15 cells from a mouse neuroblastoma × rat glioma hybrid also exhibited Aβ_42_ aggregation at the peripheral region (Supplementary Fig. S2). This indicates that the aggregation of Aβ_42_ is a ubiquitous phenomenon in living cells. Then, we evaluated the neurocytotoxic effects of Aβ_42_ and QD nanoprobes using the 3-(4, 5-dimethylthial-2-yl)-2, 5-diphenyltetrazalium bromide (MTT) assay, and confirmed that the addition of Aβ_42_ reduced cell viability (Supplementary Fig. S3). The combinations of QD or QDAβ and Aβ_42_ also exhibited almost the same result as Aβ_42_ alone. These results indicate that the addition of QDAβ had little effect on the viability of Aβ_42_-treated cells. 3D real-time imaging revealed that early aggregation at the cell periphery occurred after 8 h of incubation (Fig. 2A,B and Supplementary Movies S1 and S2). After 16 h of incubation, there was a dramatic increase in the accumulation of Aβ_42_ aggregates on the cell side. As shown in Fig. 2C, we suggested the model of Aβ_42_ aggregation at the cell periphery. The amount of aggregates increased over time. Interestingly, these 3D observations showed that Aβ_42_ aggregation was not localized on the top of the cell. The amount of Aβ_42_ aggregates surrounding the cell periphery increased over time (Fig. 2D). To clarify the mechanism by which Aβ_42_ binds to cells, cell membranes were stained with a fluorogenic membrane dye, CellBrite green (Fig. 2E). Aβ_42_ and the cell membrane colocalized, indicating that Aβ_42_ might aggregate on the cell surface by specifically interacting with the cell membrane.

### Interaction between Aβ_42_ aggregation and protrusive cell membrane

**Figure 3:**
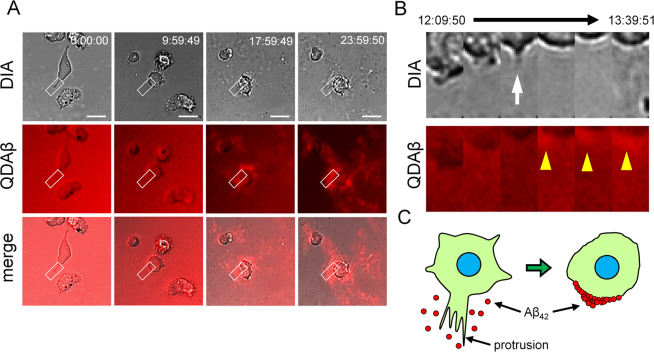
2D real-time imaging of cell protrusion and Aβ_42_ aggregation. (**A**) PC12 cells were co-incubated with 20 μM Aβ_42_ and 30 nM QDAβ, and observed by a conventional inverted microscope (DIA) and confocal microscope (QDAβ). Time series of images show the gradual steps of Aβ_42_ aggregation at the cell periphery. Bar = 20 μm. (**B**) Kymograph of cell periphery in enlarged image of panel A (white boxes) show that Aβ_42_ starts to aggregate after formation of the protrusion. The cell is localized on the top side of the panel. White arrow and yellow arrowheads indicate membrane protrusion and Aβ_42_ aggregates, respectively. (**C**) Schematic model of the Aβ_42_ aggregation around protrusions of PC12 cells.

**Figure 4:**
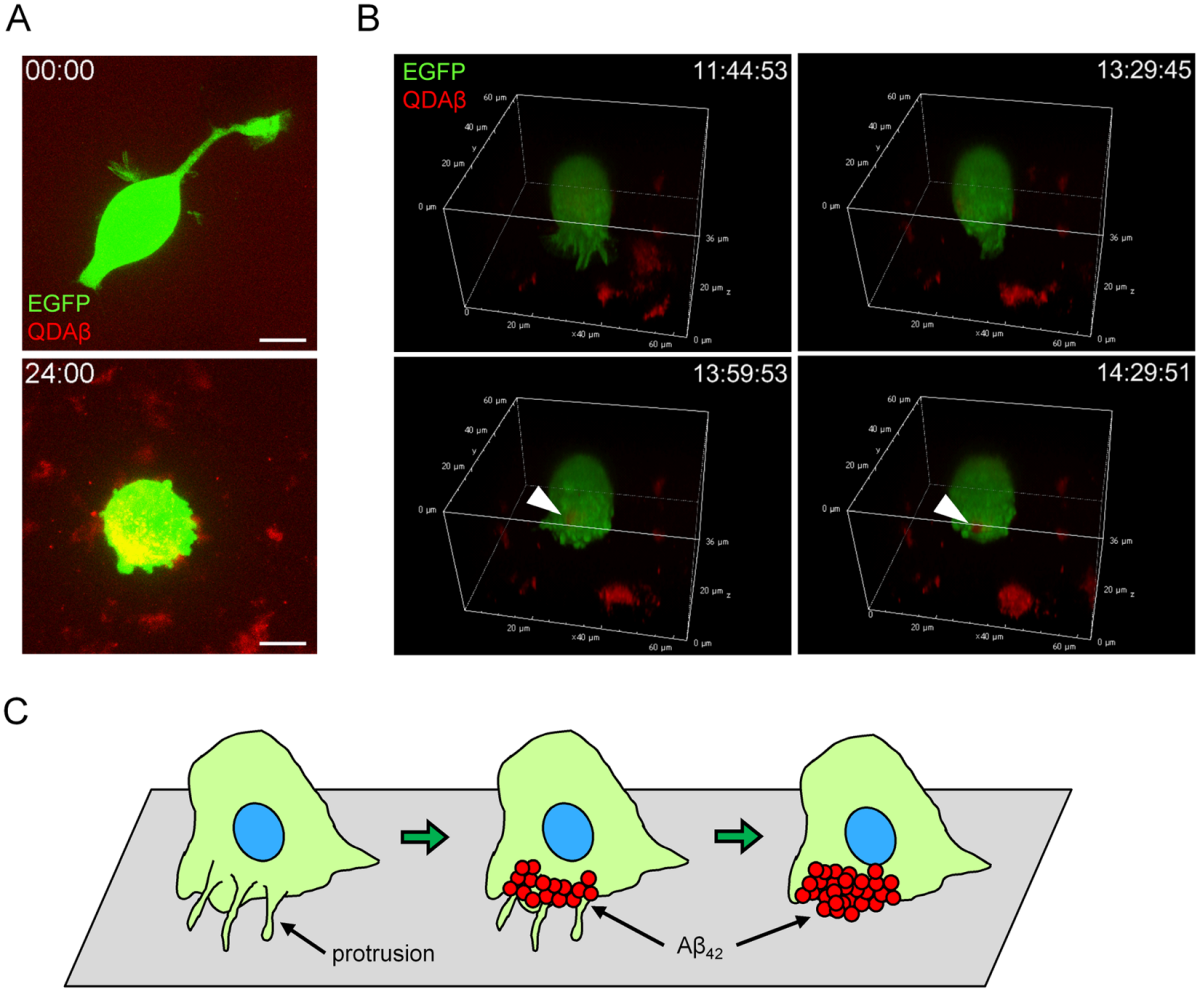
3D real-time imaging of cell protrusion and Aβ_42_ aggregation. (**A**) Image of maximum fluorescence intensity projection of 0 and 24 h time point in 3D real-time imaging. PC12 cells were co-incubated with 16 μM Aβ_42_ and 30 nM QDAβ. Aβ_42_ aggregates at the cell periphery at the 24 h time point. Bar = 10 μm. (**B**) 3D reconstruction images of Panel (A). Active protrusions formed from the cell surface at 11 h 45 min (upper left). Aβ_42_ started to aggregate in the part indicated by white arrowheads. Images were captured by a confocal microscope. (**C**) Schematic 3D model of the Aβ_42_ aggregation around protrusions of PC12 cells.

To reveal the process of Aβ_42_ aggregation at the cell’s periphery, we performed multi-channel live cell imaging using QDAβ. 2D time lapse observation clarified the formation of Aβ_42_ aggregates with movement of PC12 cell protrusion (Fig. 3A and Supplementary Movie S3). After 24 h of incubation, Aβ_42_ aggregation was observed in the region where membrane protrusions were frequently formed. A kymograph at the edge of the cell showed that Aβ_42_ aggregation first occurred around the protrusions (Fig. 3B, white arrow). Here, we suggested the model of hypotheses that active cell protrusion enhanced Aβ_42_ aggregation (Fig. 3C). In the 3D time lapse observation, the concentration of Aβ_42_ was reduced to 16 μM to facilitate analysis of Aβ_42_ dynamics on the cell membrane and PC12 cells were transiently transfected to a plasmid with enhanced green fluorescent protein (EGFP) to distinguish cell morphology (Fig. 4). Still images of a maximum fluorescence intensity projection movie showed a time-dependent increase of Aβ_42_ aggregates around the cell (Fig. 4A and Supplementary Movie S4) and the incorporation of Aβ_42_ and QDAβ to intracellular regions by phagocytosis^27^. Furthermore, still images of a 3D reconstruction movie revealed that Aβ_42_ preferentially started to aggregate at the region where membrane protrusions frequently formed (Fig. 4B and Supplementary Movie S5). In Fig. 4C, we suggested 3D model of Aβ_42_ aggregation around the PC12 cell.

**Figure 5:**
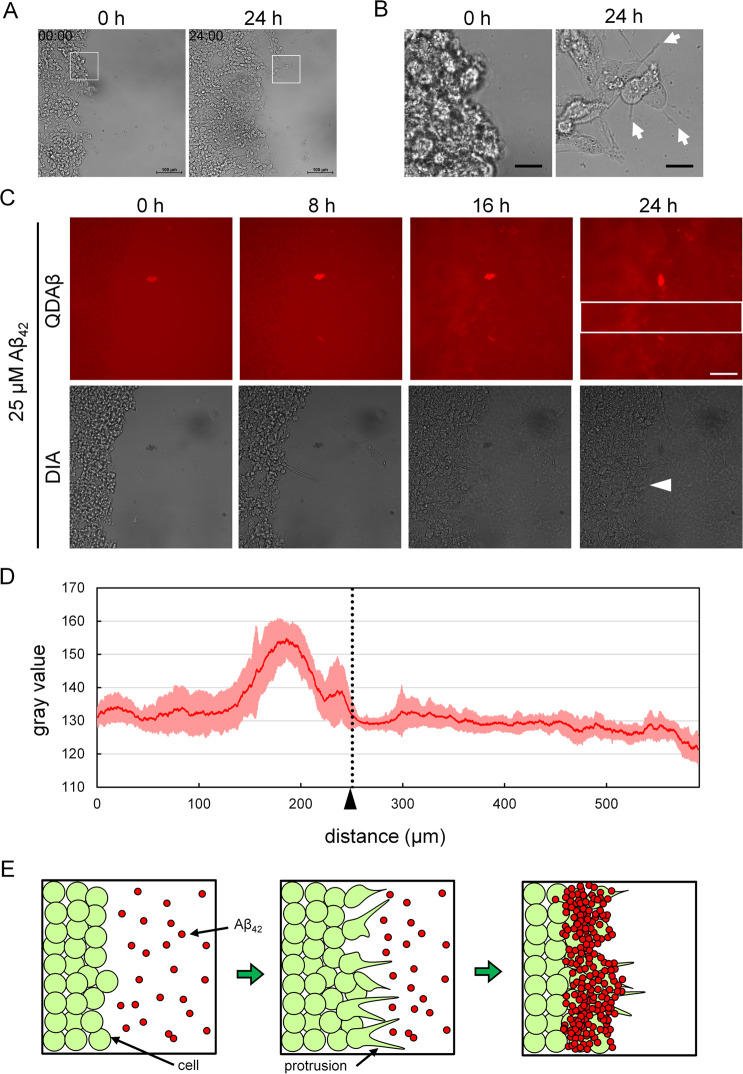
Aβ_42_ aggregation induced at edge of migrating PC12 cells. (**A**) The monolayer of PC12 cells was scratched by toothpick and defined as 0 h. Images of live cell migrated to wounded gap were captured every 10 min for 24 h. Bar = 100 μm. (**B)** Boxed region of panel A. Image of 24 h time point showed that migrating PC12 cells elongated protrusions (white arrows). Bar = 20 μm. (**C**) The monolayer of PC12 cells was scratched by toothpick. Then, PC12 cells were co-incubated with 25 μM Aβ_42_ and 30 nM QDAβ, and observed by a conventional inverted microscope. Time series of images show the gradual steps of cell migration and Aβ_42_ aggregation at wounded edge (white arrowhead). Images of live cell migrated to wounded gap were captured every 10 min for 24 h. Images of QD channel were false-colored in red using image J software. Bar = 100 μm. (**D**) Profile plot of boxed region of Panel (C) (QD channel at 24 h) indicates mean gray value per pixel. Plotted value displays a column average plot in boxed region (red line). The line was represented with error bar means ± SD (pink line). Black arrowhead and black dot line indicate the edge of cell group. (**E**) Schematic model of the Aβ_42_ aggregation at edge of migrating PC12 cells.

To elucidate whether Aβ_42_ aggregations were formed around the cell protrusion specifically, we performed wound healing assay (WHA) with Aβ_42_ and observed Aβ_42_ aggregation processes. WHA is simple and inexpensive method to evaluate the cell motility such as migration rate and directionality^31^. In this method, a confluent cell monolayer was scratched by sharpen tip. Then, it was observed closure process of artificial gap by cell motility. Wu *et al*. reported that PC12 cell showed active protrusions and recovery wounded gap during migration in this assay^32^. As shown in Fig. 5A, we confirmed PC12 cells continued to migrate toward newly created space for 24 h observation. In 24 h time point, PC12 cell exhibited active protrusions (Fig. 5B: white arrows). Incubation with Aβ_42_ and QDAβ during cell migration, we succeeded in observation of preferential formation of Aβ_42_ aggregates at the wounded edge (Fig. 5C and Supplementary Movie S6). To clarify where aggregations were occurred, we analyzed fluorescence intensity profiles along the boxed region in Fig. 5C (+Aβ_42_) and Supplementary Fig. S4 (+DMSO). Fluorescence intensity profiles indicated that Aβ_42_ treated PC12 cells showed characteristic peak around cell edge, suggesting that Aβ_42_ aggregates were formed at the wounded edge (Fig. 5D and Supplementary Fig. S4B). The aggregates showed about 100 μm width across the wounded edge. In other words, Aβ_42_ actively aggregates in the anterior group where cell motility is active (the width of a few cells). We suggested the schematic illustration of preferential aggregation at the wounded edge where frequently protrusions were formed (Fig. 5E). These results demonstrate that active membrane protrusions are essential for the promotion of Aβ_42_ aggregation at the cell periphery.

### Observed Aβ_42_ aggregation-induced apoptosis of PC12 cells

**Figure 6:**
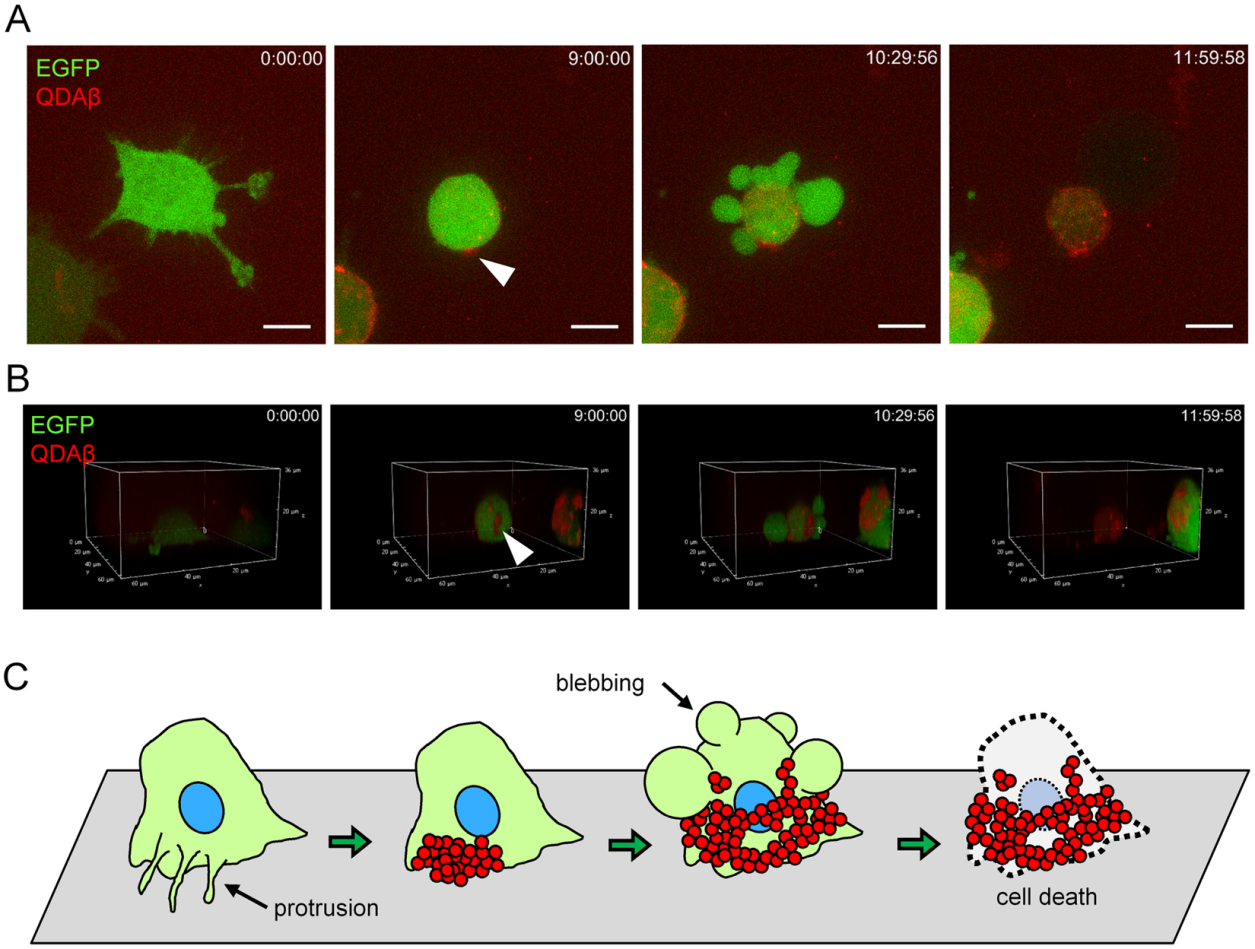
Cell death induced by Aβ_42_ aggregation. (**A**,**B**) Time series images of maximum fluorescence intensity projection (**A**) and images of 3D reconstruction (**B**) of real-time imaging. PC12 cells were co-incubated with 16 μM Aβ_42_ and 30 nM QDAβ. Note that the Aβ_42_-aggregated cell (white arrowheads) exhibited abnormal blebbing and cell death. Images were captured by a confocal microscope. After abnormal blebbing formation, cell death was suddenly induced by Aβ_42_ aggregation. Bars = 10 μm. (**C**) Schematic model of the cell death caused by Aβ_42_ aggregation around PC12 cells.

The aggregation and accumulation of Aβ_42_ on the surface of a cell membrane have various adverse effects on cellular physiological homeostasis. Actually, human neuroblastoma, SH-SY5Y cells exhibiting the accumulation of Aβ_42_ aggregates undergo apoptosis^33^. As shown in Fig. 6A,B and Supplementary Movie S7, we demonstrated that Aβ_42_ aggregation on the cell surface abruptly caused cell death. Additionally, we found that aggregates of Aβ_42_ cause abnormal cell morphology and the flow of cytoplasmic components out of the cell due to destruction of the cell membrane (Supplementary Fig. S5). Furthermore, we found that Aβ_42_ aggregation led to defects in maintenance of nuclear morphology (Supplementary Fig. S6). These phenomena are considered to be important processes in the mechanism of cell death caused by Aβ_42_ aggregation. Interestingly, after cell death, Aβ_42_ aggregates, which accumulated on the cell surface, remained there. As shown in Fig. 6 (time point at 11 h 59 min), even after the membrane blebbed and the cytoplasm flowed out, the Aβ_42_ aggregates maintained a rough cell morphology, like a “husk”. We suggested the schematic illustration of cell death caused by Aβ_42_ aggregations (Fig. 6C). Furthermore, additional Aβ_42_ aggregation occurred and continued to grow, so the basis of this “husk” consists of Aβ_42_ aggregates (Supplementary Movie S7: time point at 14 h 14 min).

### F-actin polymerization enhanced Aβ_42_ aggregation on the cell surface via protrusion regulation

**Figure 7:**
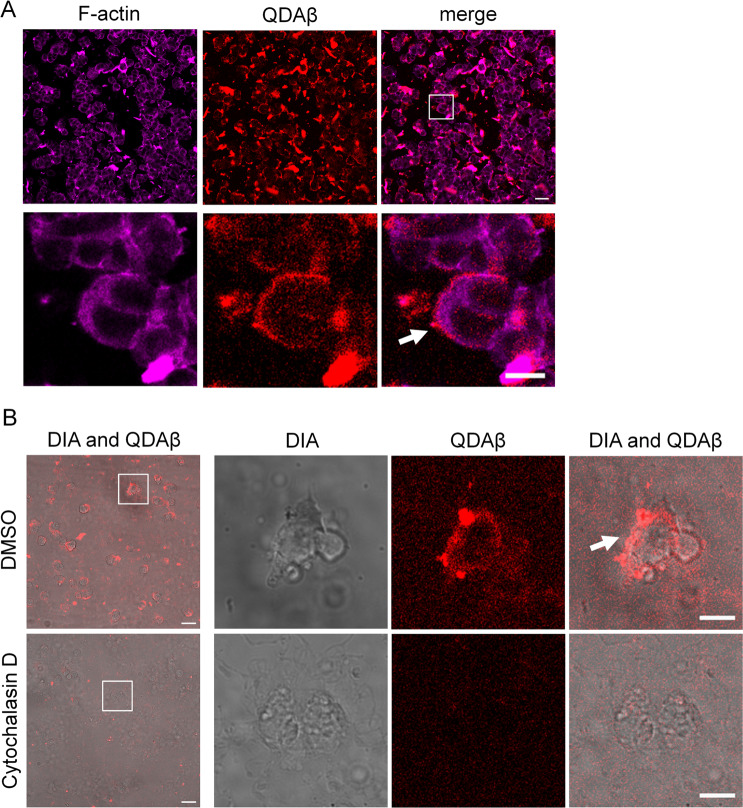
F-actin polymerization-dependent cell membrane movement is required for Aβ_42_ aggregation on the cell surface. (**A**) PC12 cells were co-incubated with 20 μM Aβ_42_ and 30 nM QDAβ (red) for 24 h and were stained with Alexa 647-phalloidin (magenta), and then observed by a confocal microscope. Bar = 50 μm. Bottom panels indicates enlarged images of boxed region in the top panels. Bar = 20 μm. The white arrow points to colocalization of Aβ_42_ aggregates and F-actin accumulation in the cell periphery region. (**B**) PC12 cells were treated with DMSO (control) or 20 μg/ml cytochalasin D for 24 h and then co-incubated with 20 μM Aβ_42_ and 30 nM QDAβ (red) for 24 h. Bar = 50 μm. Images were captured using a confocal microscope (QDAβ) and a conventional inverted microscope (DIA). Right three panels indicate enlarged images of the boxed region in the left panel. Cytochalasin D inhibited Aβ_42_ aggregation at the cell periphery. Bar = 20 μm.

Here, we hypothesized that cytoskeletal actin, which is essential for the control of cell morphology and cell motility, is involved in the mechanism of Aβ_42_ aggregation around cells. To investigate the role of actin in protrusion-dependent Aβ_42_ aggregation, we visualized F-actin of PC12 cells using Alexa 647-phalloidin. Aβ_42_ and F-actin colocalized at the cell periphery (Fig. 7A). Many Aβ_42_ aggregates were localized in the region where F-actin had accumulated. To examine whether actin polymerization avidity in protrusions is necessary for Aβ_42_ aggregation, we inhibited actin polymerization of cells using 20 μg/ml cytochalasin D, which is an actin polymerization inhibitor that caps the barbed end of F-actin^34^. Before the experiment, we analyzed the effect of cytochalasin D on Aβ_42_ aggregation using MSHTS methods^28,29^. In MSHTS method, we quantified the amount of Aβ_42_ aggregates from standard deviation (SD) values of fluorescence intensity of each pixel. Addition of cytochalasin D (0.16-100 μg/ml) did not change the SD value compared to control sample (Supplementary Fig. S7), indicating that Aβ_42_ aggregation was not directly inhibited by 100 μg/ml cytochalasin D. Then, we confirmed that 20 μg/ml of cytochalasin D treatment suppressed Aβ_42_ aggregation around cells (Fig. 7B). These results indicate that the formation of F-actin polymerization-dependent protrusions is required for the preferential aggregation of Aβ_42_ at the cell periphery.

## Discussion

In this study, by adding Aβ_42_ and QDAβ to a culture model neuron, we succeeded in real-time imaging of the aggregation process of Aβ_42_ around cells. It was revealed that the aggregation of Aβ_42_ is not uniform on the cell surface and that preferential aggregation is promoted at the cell periphery where cell protrusions are intensely formed. We also demonstrated that actin polymerization-dependent cell motility is responsible for the promotion of Aβ_42_ aggregation at the peripheral region of PC12 cells.

Lu *et al*. reported the gradual aggregation of Aβ that exists inside culture cells and defined the assembly of Aβ fibrils as an aggresome that grows until several micrometers in diameter^10^. Here, we also visualized extracellular aggregation. We confirmed that Aβ_42_ aggregates formed a unit several micrometers in diameter and that these Aβ_42_ units interacted with each other, then aggregated in solution (M. Kuragano *et al*., manuscript in preparation). It is possible that Aβ_42_ aggregates might exhibit an upper size limit of several micrometers. By using a super-resolution microscope, new insight about classification of the process of aggregation at the cell periphery might be obtained.

**Figure 8:**
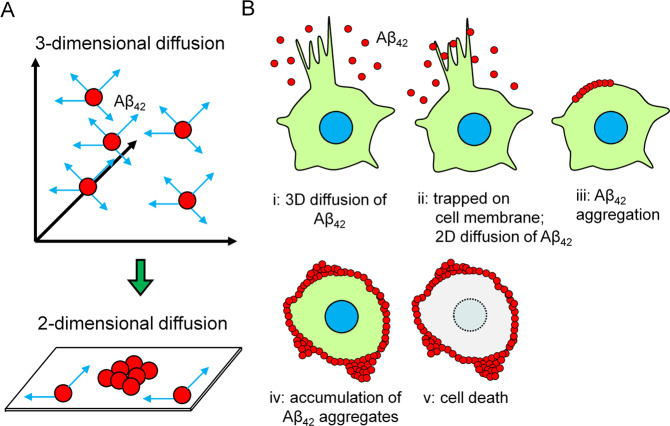
Schematic model of novel Aβ_42_ aggregation process on the cell surface. (**A**) Transition of the Aβ_42_ movement state from three-dimensional to two-dimensional diffusion. Due to the frequency of collisions between each Aβ_42_ monomer on the cell membrane surface, aggregation is thought to increase there. (**B**) Relationship between the formation of cell protrusion and promotion of Aβ_42_ aggregation. The frequency of collisions between each Aβ_42_ monomer on the cell membrane might increase in the area where active protrusions formed, more than in the static region. Therefore, it is thought that Aβ_42_ aggregate formation is particularly promoted at the cell edge where movement is active.

Recently, it was reported that the aggregation of Aβ is promoted under the presence of surfactants, such as micelles, near the cell membrane^16,35^. Wakabayashi *et al*. reported that gangliosides present in sphingolipids and cholesterol-rich domains called “lipid rafts” on cell membranes function as scaffolds for Aβ aggregation^15^. Here, we hypothesized that the diffusion of Aβ_42_ transits from a three-dimensional area in culture medium to a two-dimensional plane on the surface of the cell membrane (Fig. 8A) and that the intense cell membrane protrusions driven by actin polymerization dramatically increase the frequency of collisions among Aβ_42_ molecules at the cell surface (Fig. 8B), which promotes additional Aβ_42_ aggregation. Observation of the maturation of early Aβ_42_ aggregates *in vitro* also supports the possibility that the aggregation nucleus initially formed on the cell surface, incorporated new Aβ_42_, and exhibited the growth of aggregates (Supplementary Fig. S8 and Supplementary Movie S8).

We also showed that Aβ_42_ aggregates at the cell surface can cause cell death. Neuronal death is considered to be triggered by promoting the formation of Aβ_42_ aggregates on the cell membrane. In fact, apoptosis of SH-SY5Y cells, was induced by Aβ fibrillation^33^. After abnormal morphological changes of the cell and nucleus, the cell membrane was disrupted, then neuronal cells died. Moreover, we found that the reduced cell membrane plasticity caused by Aβ_42_ accumulation may be involved in the expression of its neurocytotoxicity^36,37^. Remarkably, Aβ_42_ aggregates on the surface of PC12 cells remained there, even after cell death. After frequent membrane blebbing and spillage of the cellular components due to cell death, the aggregated Aβ_42_ continued to stay in place as if it had left the cell outline. This “husk” also seems to play the function of an aggregation nucleus, i.e., new aggregates accumulated on this “husk”. The amyloid cascade hypothesis suggested that the emergence of an amyloid plaque (senile plaque) is major feature of the expression of AD^2^. Although the real-time imaging of plaque formation in local neuronal tissue of transgenic mice was observed using multiphoton microscopy^38^, details of the processes and molecular mechanism underlying its formation are still obscure^39^. In addition to the newly formed plaques in the microenvironment near the neuronal cells^38^, we hypothesized that Aβ_42_ aggregation remaining around dead cells might mature into amyloid plaques. Actually, it was reported that the size-order of amyloid plaques is about the same as neuronal cells and that amyloid plaques consist of proteins, carbohydrates, nucleic acids, lipids, and metal ions^40,41^. Among these major components, lipid rafts on the surface of PC12 cells are known scaffolds of Aβ_42_ aggregation, as mentioned above^15^. It is possible that the debris of dead cells arising from Aβ_42_ neurocytotoxicity accelerate the progression of AD.

F-actin is necessary to regulate neuronal morphology^20^ and maintain spine plasticity^42^. SynGAP, a GTPase-activating protein, is the main regulator of actin dynamics of the dendritic spine and is involved with cofilin, which caused disassembly of F-actin by its serving activity^43^. Drebrin is an F-actin-binding protein and is highly expressed in brain tissue^44^. In normal spines, drebrin protects F-actin from serving by cofilin activity. In other words, the drebrin-actin complex exhibits an important function by maintaining spine morphology. Separately, cofilin caused the disappearance of F-actin polymerization in the AD spine^45^. However, we demonstrated that Aβ_42_-treated PC12 cells accumulated F-actin in the cortical region (Fig. 7A). It was reported that cofilin mediated Aβ-induced apoptosis^46^. Our results imply that the cofilin-independent apoptosis pathway was stimulated by the aggregation of Aβ_42_ on the cell surface. In fact, it was reported that F-actin acts as a sensor of apoptosis^47^ and that the apoptosis of endothelial cells was caused by stimulation of TNF-α through changes in the actin cytoskeleton and an increase in phosphorylation of the regulatory light chain of non-muscle myosin II^48^. It is possible that Aβ_42_ aggregation directly suppresses flexibility of the cell membrane and decreases cell plasticity. In addition, the F-actin cytoskeletal fractions, which are supposed to be used for cell membrane protrusion, are thought to have nowhere to localize, allowing them to start to accumulate under the membrane. Consequently, the apoptosis pathway might be induced. It is essential to consider in the future whether the excessive accumulation of actin, which is induced by Aβ_42_, may affect myosin II activity. A well-known structure in the brain, the dendritic spine, protrudes from the dendrite of a nerve cell^49^. Changes in the number and shape of these structures, which play a role in receiving information from most excitatory synapses in the brain, are thought to be involved in the mechanism of neuroplasticity^49,50^. Dendritic spines are generally classified into two sizes, small and large spines, which exhibit different properties^50,51^. Particularly, immature small spines, with filopodia-like structures, exhibit more active movement and a shorter lifetime than large spines^49^. As shown in Figs. 3–5, dynamic membrane movement promoted Aβ_42_ aggregation around cells. It is possible that Aβ_42_ aggregation occurred preferentially on the surface of small spines than on large spines in the brain. In other words, it is reasonable to expect that small spines are susceptible to neurocytotoxicity due to Aβ_42_ aggregation. Although large spines have more F-actin than small spines in proportion to their surface area, its motility is lower than that of small spines. Currently, we are working on elucidating the molecular mechanism of Aβ_42_ aggregation-dependent cell death through F-actin accumulation.

## Materials and Methods

### Cell culture

Rat adrenal pheochromocytoma, PC12 cells, were obtained from the JCRB Cell Bank (Japan). Mouse neuroblastoma × rat glioma, NG108-15 cells, were a kind gift by Prof. Hiroyuki Nakagawa (Fukuoka University). PC12 cells were maintained in RPMI (Gibco/Life Technologies, USA) supplemented with 5% fetal bovine serum (FBS) (Gibco/Life Technologies) and 10% horse serum (HS) (Gibco/Life Technologies) or DMEM (Wako, Japan) supplemented with 10% FBS. NG108-15 cells were maintained in DMEM supplemented with 10% FBS. All culture media were supplemented with 100 U/mL penicillin and 100 μg/mL streptomycin (Wako). Cells were cultured at 37 °C in humidified air containing 5% CO_2_.

### Reagents

Poly-D-Lysine was purchased from Sigma-Aldrich (USA). Human Aβ_42_ (4349-v; Peptide Institute, Japan) and Cys-conjugated Aβ_40_ (23519; Anaspec, USA) were purchased commercially. Nerve growth factor was purchased from Cosmo Bio (Japan). Alexa 647-phalloidin and Hoechst 33342 were purchased from Invitrogen (USA). CellBrite were purchased from Biotium (USA).

### Preparation of QDAβ nanoprobes

QDAβ nanoprobe was prepared using QD-PEG-NH_2_ (Qdot 655 ITK Amino (PEG) Quantum dot; Q21521MP or Qdot 605 ITK Amino (PEG) Quantum dot; Q21501MP, Thermo Fisher Scientific, USA) according to our previous reports^28–30^. The QDAβ nanoprobe was prepared as follows: 10 µM QD-PEG-NH_2_ was first reacted with 1 mM sulfo-EMCS (22307; Pierce/Thermo Fisher Scientific) in PBS for 1 h at room temperature. After quenching and eliminating unreacted sulfo-EMCS, the QD-PEG-NH_2_-bound sulfo-EMCS was reacted with 74 μM Cys-conjugated Aβ_40_ for 1 h at room temperature. The concentration of QDAβ was determined by comparing absorbance at 350 nm to unlabeled QD-PEG-NH_2_.

### MSHTS system

The SD values in existence of cytochalasin D were determined by a modified MSHTS system, as was described in our previous reports^28–30^. More specifically, various concentrations of cytochalasin D, 25 μM Aβ_42_ and 25 nM QDAβ in PBS were incubated in a 1536-well plate (782096, Greiner Bio-One, Austria) at 37 °C for 24 h. The QDAβ-Aβ_42_ aggregates that formed in each well were observed by an inverted fluorescence microscope (TE2000, Nikon) equipped with a color CCD camera (DP72, Olympus) and an objective lens (Plan Fluor 4×/0.13 PhL DL, Nikon). SD values of fluorescence intensities of 40,000 pixels (200 × 200 pixels) around the central region of each well were measured by Image J software (NIH, USA).

### MTT assay

PC12 cells were plated at 2.0 × 10^4^ cells in poly-D-lysine-coated 96-well plates (IWAKI, Japan) and incubated for 24 h. After incubation, cells were treated with 4.5 ng/ml NGF and further incubated for 24 h. Then, cells were treated with QD, QDAβ, and Aβ_42_ and cells were incubated for a further 24 h. Then, cells were treated with 1.2 mM MTT and incubated for 4 h. After incubation, the supernatant of each well was removed and then crystals of formazan were dissolved in 10% SDS/0.01 M HCl solution. After overnight incubation, absorbance intensity (570 nm) was measured in a 96-well plate. Cell viability was assessed as a percentage relative to DMSO-treated cells.

### Transfection

pEGFP was a kind gift from Assoc. Prof. Masayuki Takahashi (Hokkaido University). Cells were transfected with plasmid DNA using Superfect Transfection Reagent (Qiagen, Germany) in DMEM according to the manufacturer’s protocol. Transfected cells were replated onto a glass-bottom 96-well plate (Corning, USA) precoated with 0.1 mg/mL poly-D-lysine for live cell imaging.

### time-lapse observation

For single cell motility observation, time-lapse images were captured with an inverted microscope (Ti-E; Nikon, Japan) equipped with a color CMOS camera (DS-Ri2; Nikon) and an objective lens (PlanApo λ 20×/0.75 NA; Nikon). During observation, the cells were maintained in DMEM/F12 (1:1) (Gibco/Life Technologies) supplemented with 10% FBS and warmed in a chamber set at 37 °C. Images were captured every 10 min and analyzed using NIS-Elements C software (Nikon). For WHA, PC12 cells were plated at 10 × 10^4^ cells in a glass-bottom 96-well plate precoated with 0.1 mg/mL poly-D-lysine. Cells were incubated for 24 h. After incubation, cells were treated with 45 ng/ml NGF and further incubated for 24 h. Then, after removal of culture medium, cell monolayer was scratched by toothpick and defined as 0 h. Then, the wells were filled by DMEM/F12 (1:1) supplemented with 10% FBS and 45 ng/ml NGF. This new medium included 1% DMSO or 25 μM Aβ_42_ and 30 nM QDAβ. Time lapse observations were performed in the same way as above described. Fluorescence images were captured as 8-bit grayscale images. All images were adjusted by a macro program so that average intensity became 50% according to our previous report method^29^. The mean gray value per pixel of Aβ_42_ aggregates was measured by the plot profile, the function of Image J software.

### Confocal laser scanning microscopy

Z-stacks images were captured using an inverted microscope (Ti-E) and a confocal laser microscope system (C2 Plus; Nikon) equipped with an objective lens (PlanApo λ 20×/0.75 NA; Nikon, Plan Apo λ 100×/1.45 NA Oil; Nikon). Cells were maintained in RPMI supplemented with 5% FBS and 10% HS and warmed in a chamber set at 37 °C (INUBTF-WSKM-B13I; Tokai Hit) during observation. Images were captured and analyzed using NIS-Elements C software. For 3D time-lapse imaging, cells were maintained in DMEM/F12 (1:1) supplemented with 10% FBS and 4.5 ng/ml NGF, and warmed in a chamber set at 37 °C during observation. EGFP and QD were excited by a laser at 488 and 561 nm, respectively. Z-stack images were captured every 15 min and were oversampled by taking 1000 nm z-steps between acquired images and were analyzed using NIS-Elements C software. For observation of Aβ_42_ aggregation by the MSHTS system, 25 μM Aβ_42_ in and 25 nM QDAβ, PBS containing 5% EtOH and 3% DMSO were incubated in a 1536-well plate (782096; Greiner) warmed in a chamber set at 37 °C. Z-stack images were captured every 3 min and were oversampled by taking 2500 nm z-steps between acquired images and were analyzed using NIS-Elements C software.

## Supplementary information

Supplementary information.

Supplementary information 2.

Supplementary information 3.

Supplementary information 4.

Supplementary information 5.

Supplementary information 6.

Supplementary information 7.

Supplementary information 8.

Supplementary information 9.
