# Supplementary material for: Three-dimensional real time imaging of amyloid β aggregation on living cells
Source: Sci Rep. 2020 Jun 16;10:9742. doi: 10.1038/s41598-020-66129-z (PMC7297742; doi:10.1038/s41598-020-66129-z)
Supplement: Supplementary file 9 — Supplementary information 9. [file 41598_2020_66129_MOESM9_ESM.pdf]

Supplementary information

Three-dimensional real time imaging of amyloid β aggregation on living cells

Masahiro Kuragano, Ryota Yamashita, Yusaku Chikai, Ryota Kitamura, and Kiyotaka Tokuraku
Graduate School of Engineering, Muroran Institute of Technology, 27-1 Mizumoto, Muroran 050-8585, Japan

*Corresponding author: Kiyotaka Tokuraku Ph. D., Graduate School of Engineering, Muroran Institute of Technology, 27-1 Mizumoto-cho, Muroran 050-8585, Japan, Telephone: +81-143-46-5721, Fax: +81-143-46-5701, e-mail: tokuraku@mmm.muroran-it.ac.jp

Supplementary Figures

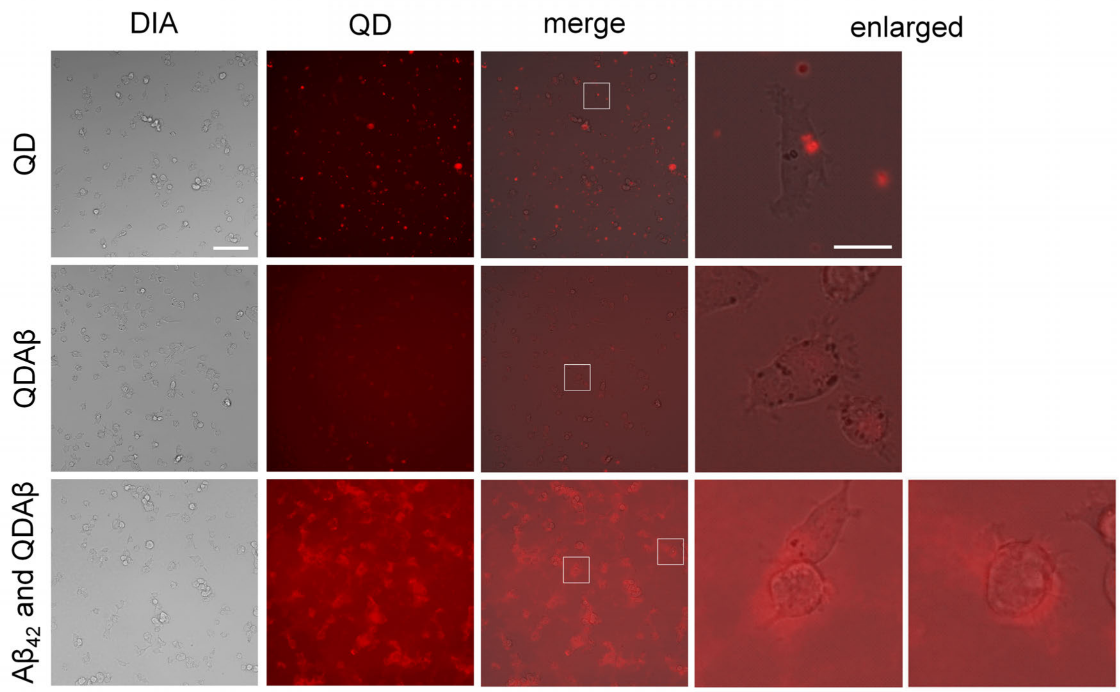

Supplementary Figure S1. Localization of QD and QDAβ on PC12 cells.

PC12 cells were co-incubated with 30 nM QD, 30 nM QDAβ, and 20 μM Aβ_{42} and QDAβ for 24 h. Note that no aggregates formed in only QD or QDAβ conditions. Bar = 100 μm. Enlarged images indicate boxed region in each condition. Bar = 20 μm. Images were captured using a conventional fluorescence microscope.

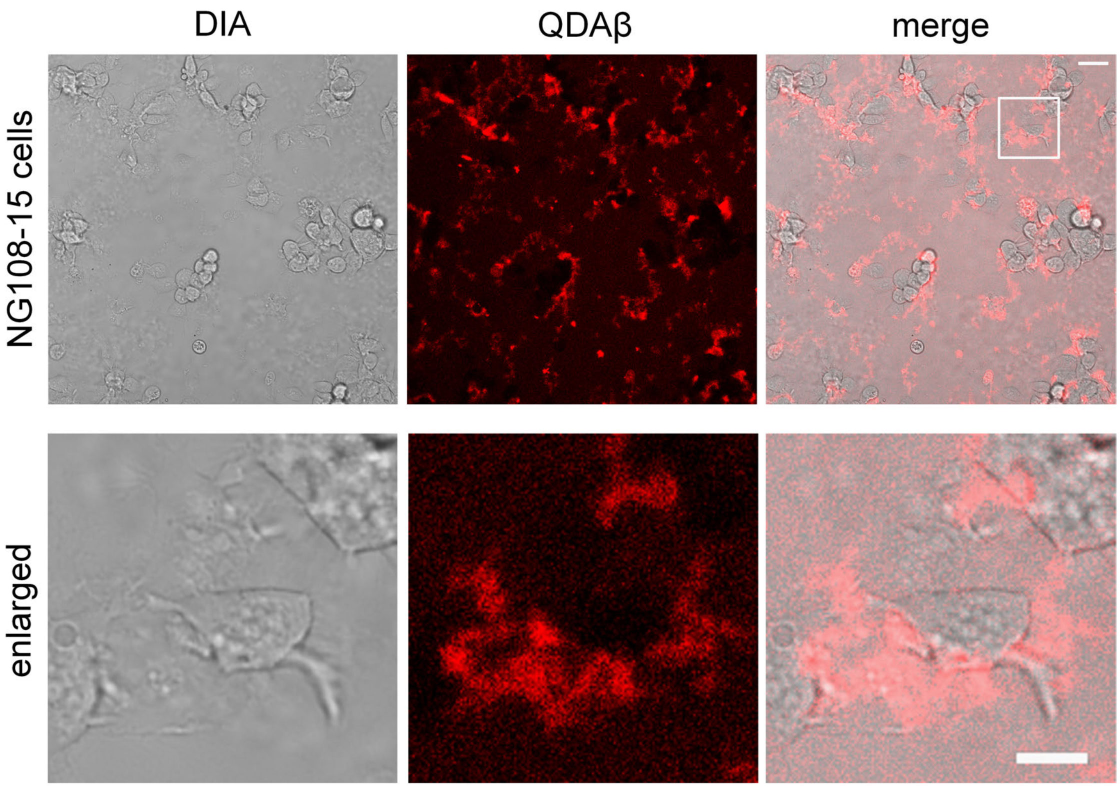

Supplementary Figure S2. Aβ_{42} aggregation at the periphery of NG108-15 cells. NG108-15 cells were co-incubated with 20 µM Aβ_{42} and 30 nM QDAβ. Aggregates of Aβ_{42} formed around cells. Bar = 50 µm. Bottom panel indicates boxed region of top panel. Bar = 20 µm. Images were captured using a confocal microscope (QDAβ) and a conventional inverted microscope (DIA).

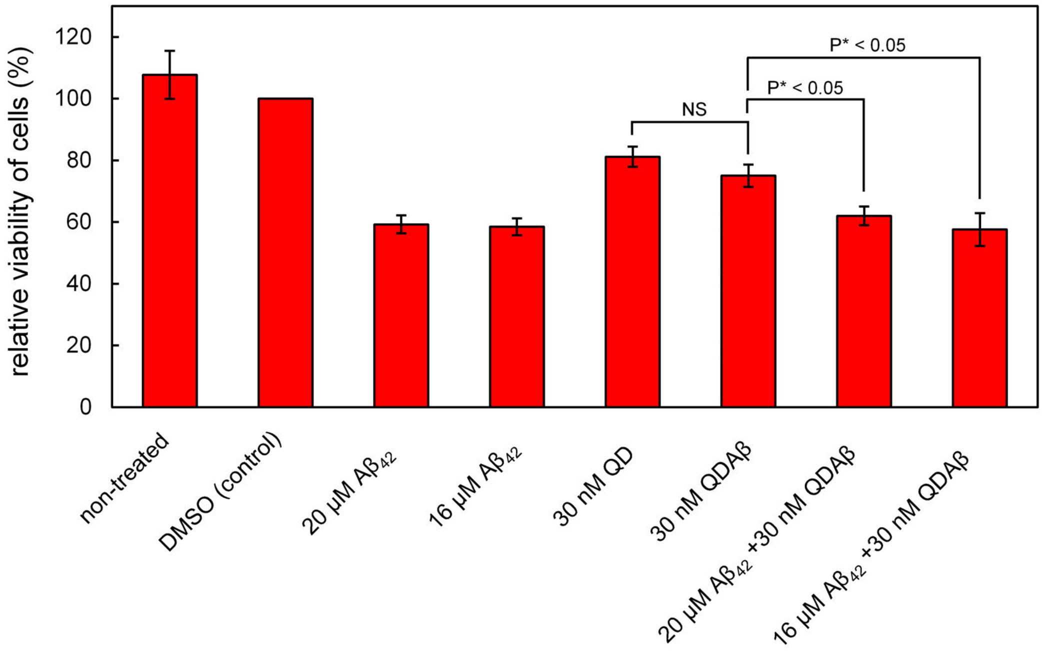

Supplementary Figure S3. Effects of Aβ_{42} and QD nanoprobes in the viability of PC12 cells.

The viability of PC12 cells was measured using the MTT assay. The combination of QD or QDAβ and Aβ_{42} did not change the viability of Aβ_{42}-treated cells. The viability of cells is a percentage relative to DMSO-treated cells (control). Data represent the mean ± SD from values of three wells. Student's *t*-test, $^{*}p < 0.05$.

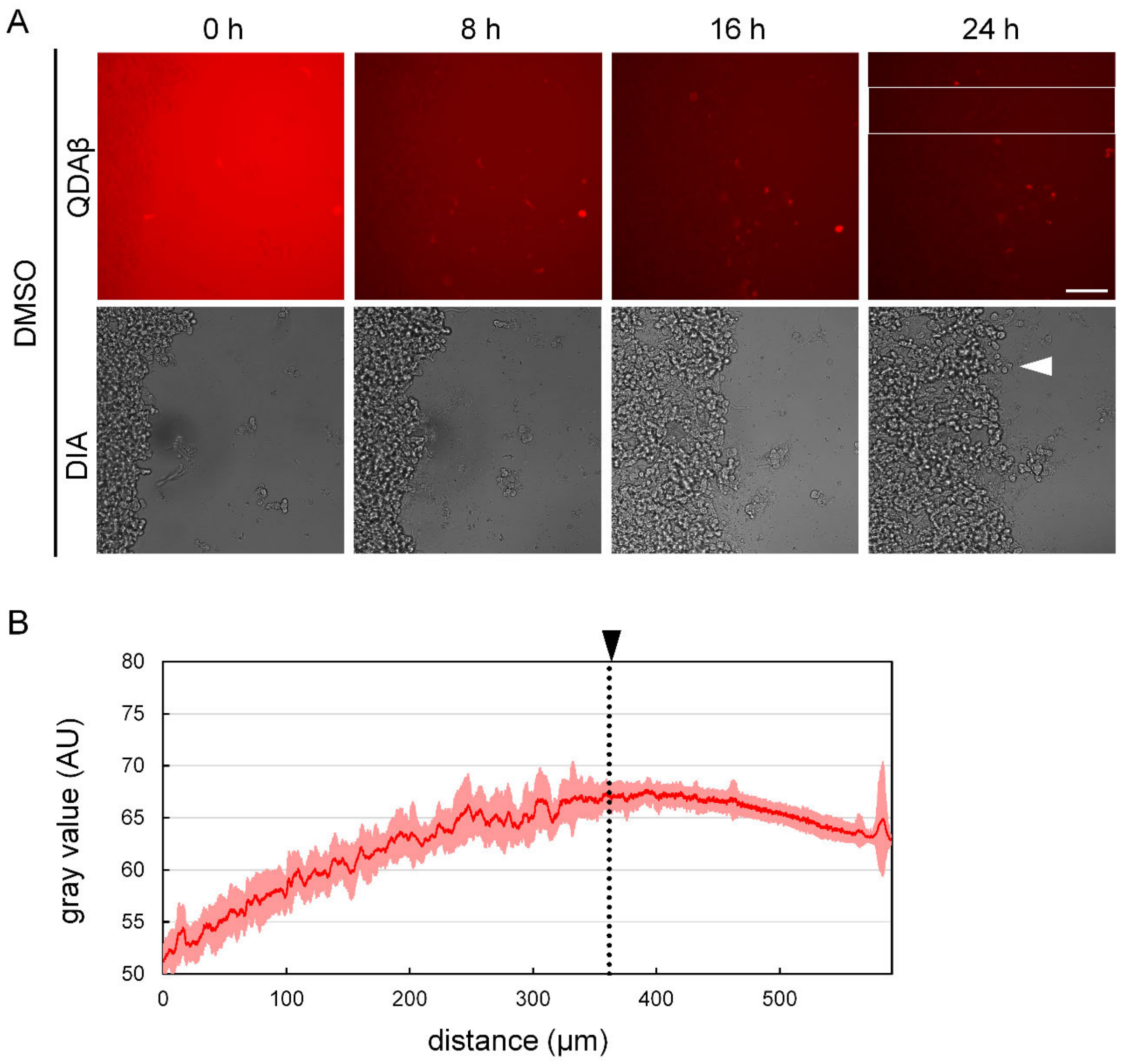

Supplementary Figure S4. Migration of DMSO treated PC12 cells during WHA. (A) The monolayer of PC12 cells was scratched by toothpick. Then, PC12 cells were co-incubated with 1% DMSO, and observed by a conventional inverted microscope. Time series of images show the gradual steps of cell migration and Aβ_{42} aggregation at wounded edge (white arrowhead). Images of live cell migrated to wounded gap were captured every 10 min for 24 h. Profile plot of boxed region at 24 h time point (QD channel) were displayed in Fig. 5D. Bar = 100 μm. (B) Profile plot of boxed region of panel A (QD channel at 24 h) indicates mean gray value per pixel. Plotted value displays a column average plot in boxed region. The line was represented with error bar mean standard deviation value (pink line). Black arrowhead and black dot line indicate the edge of wound.

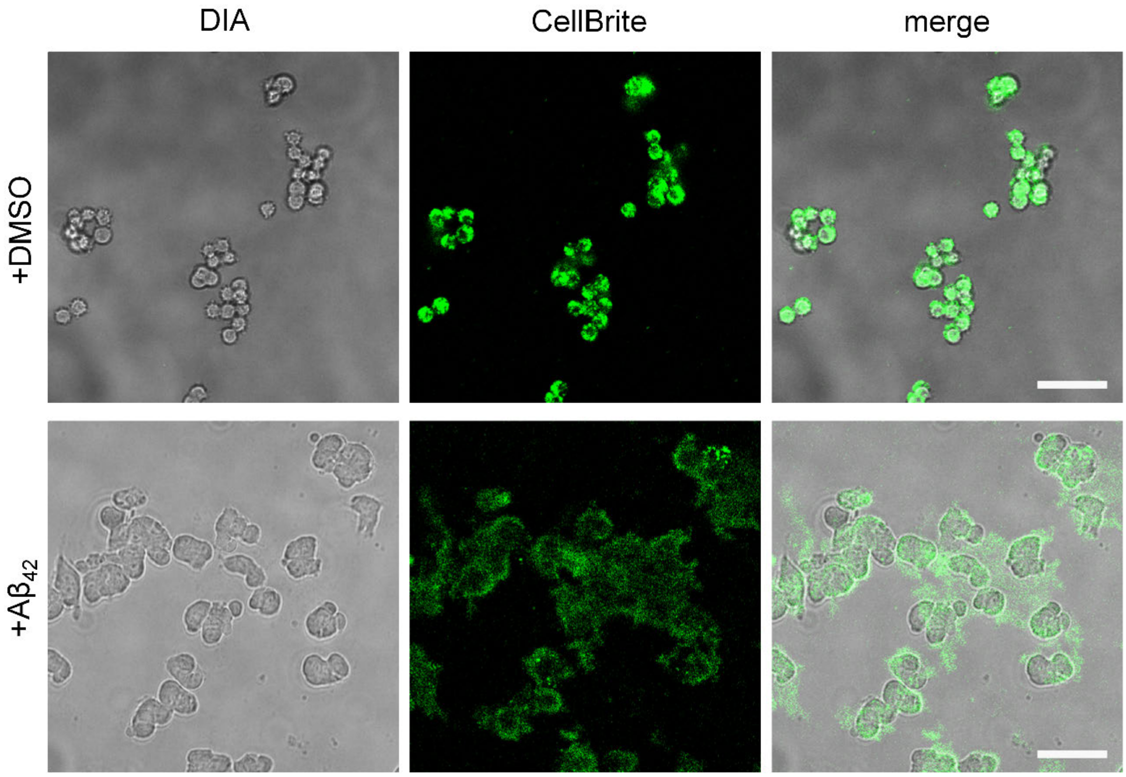

Supplementary Figure S5. Aβ_{42} treatment destroys cell membranes.

PC12 cells were coincubated with DMSO (control) or 20 μM Aβ_{42} and were stained with CellBrite green (green). Note that the cell membrane is broken by Aβ_{42} aggregation, and cell components flow out of cells. Images were captured using a confocal microscope (CellBrite) and a conventional inverted microscope (DIA). Bars = 50 μm.

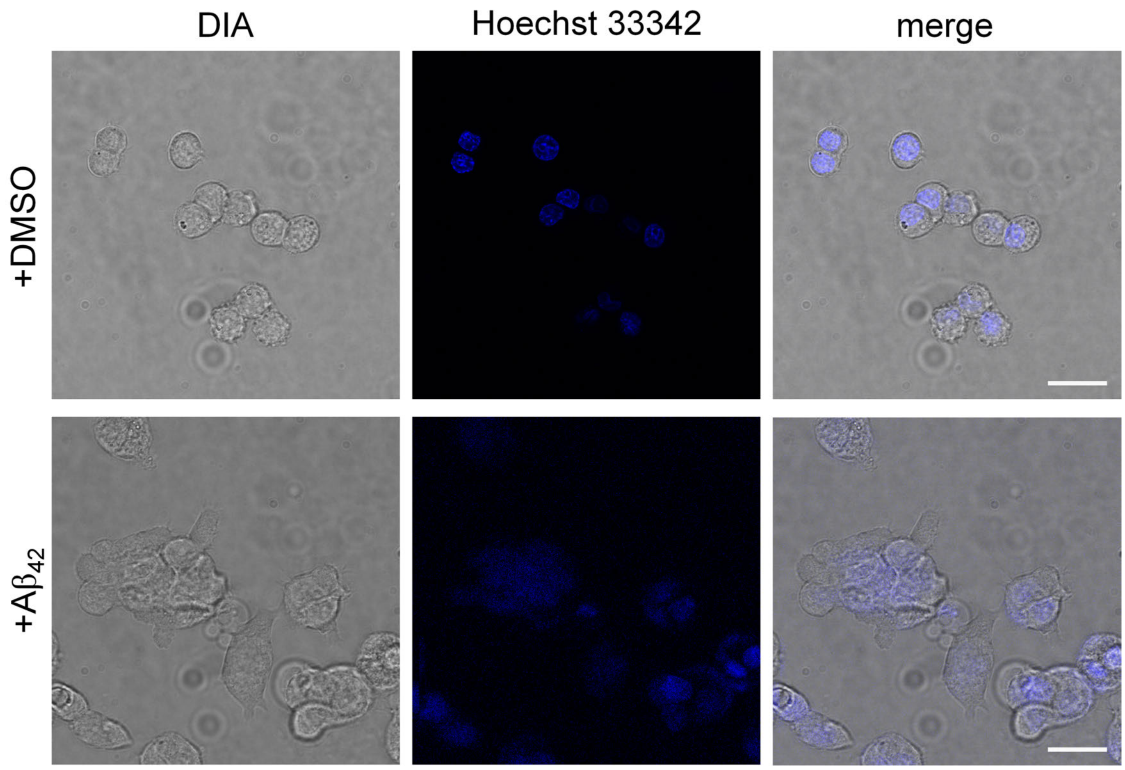

Supplementary Figure S6. Aβ_{42} treatment caused defects of the nucleus.

PC12 cells were coincubated with DMSO (control) or 20 μM Aβ_{42} and were stained with Hoechst 33342 (blue). Note that the nucleus swelled and showed abnormal morphology. Images were captured using a confocal microscope (Hoechst 33342) and a conventional inverted microscope (DIA). Bars = 50 μm.

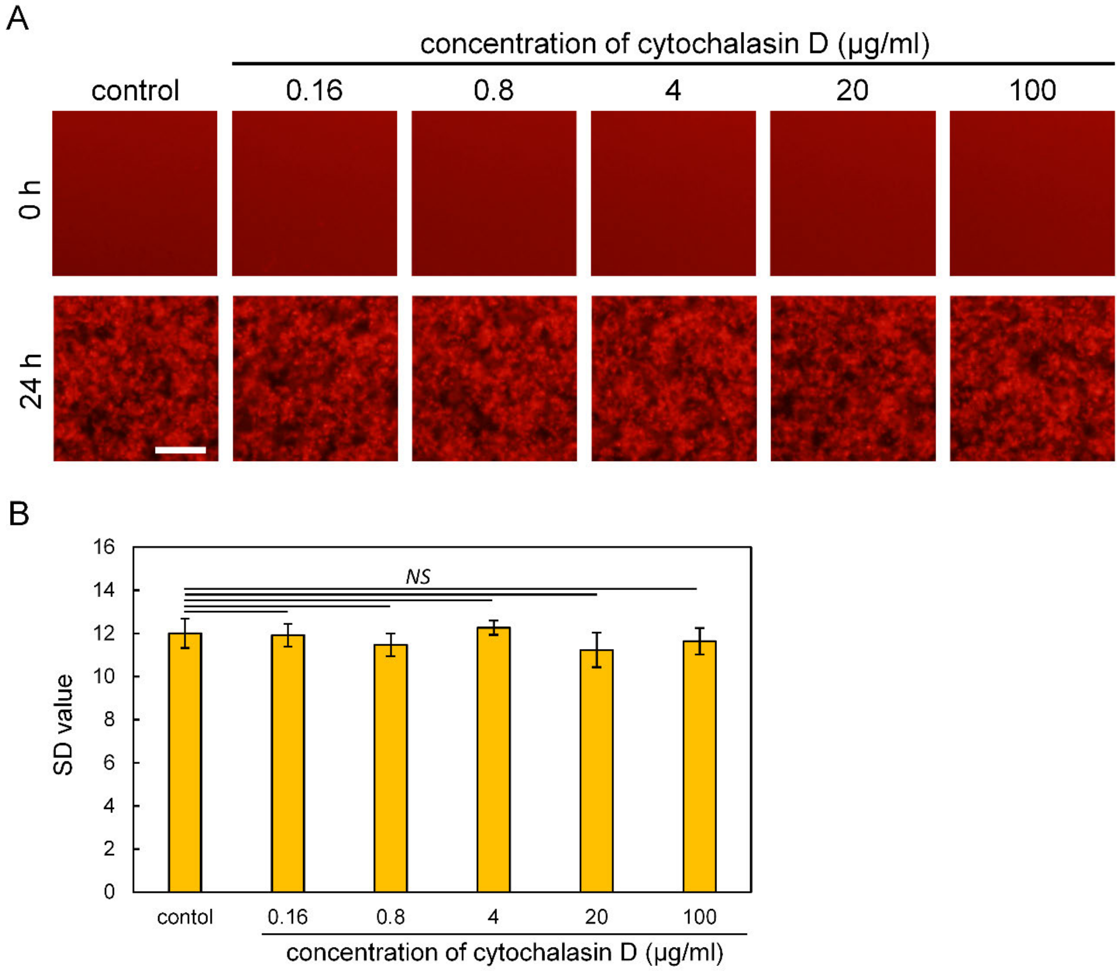

Supplementary Figure S7. Cytochalasin D did not inhibit Aβ_{42} aggregation.

(A) 25 μM Aβ_{42} and 25 nM QDAβ were incubated with various concentrations (0.16 ~ 100 μg/ml) of cytochalasin D at 37 ℃ for 24 h. Top and bottom panel indicate 0 and 24 h time point, respectively. Images were captured using a conventional fluorescence microscope. Bar = 100 μm. (B) SD values of fluorescence intensity of each pixel, which correlates with the amount of Aβ_{42} aggregates, in the presence of various concentrations of cytochalasin D. Data represent the mean ± SD from values of three wells. There were no significant differences among the groups (Student's *t*-test).

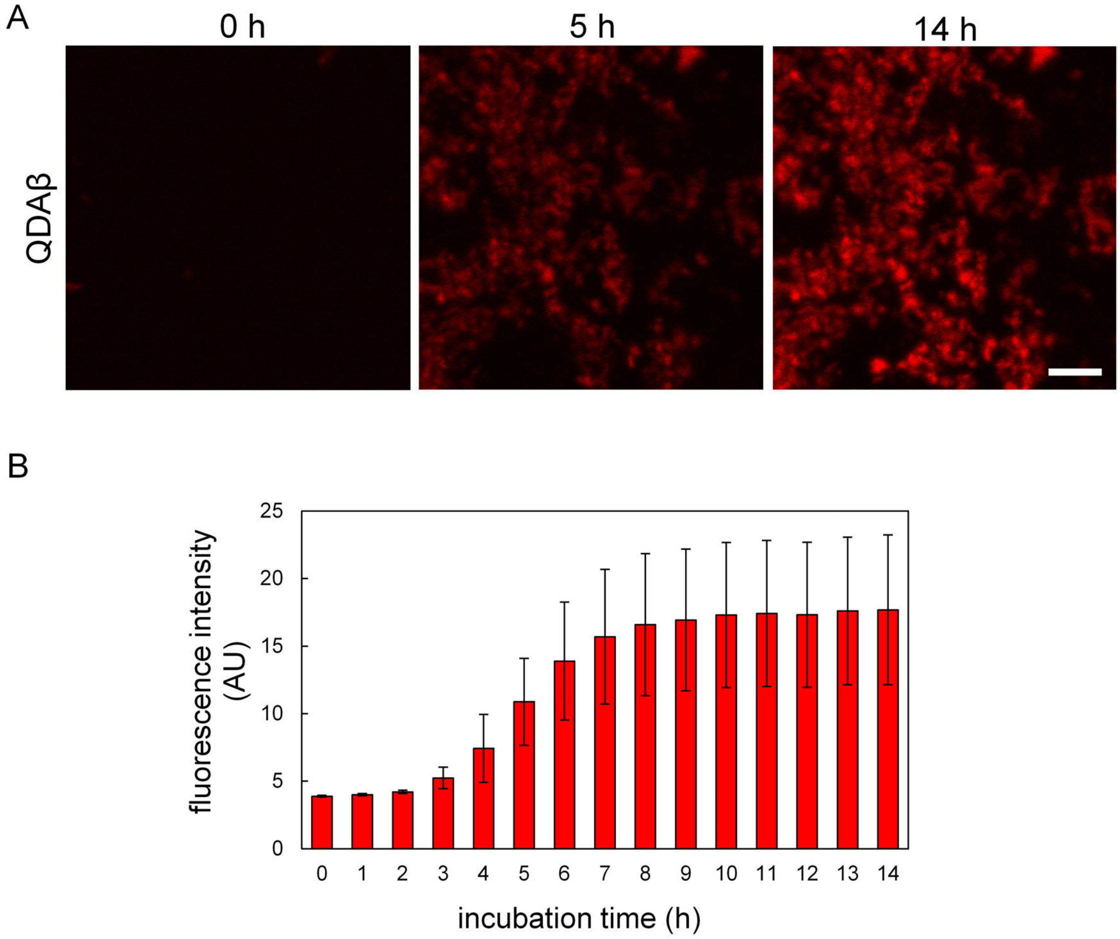

Supplementary Figure S8. Maturation of Aβ_{42} aggregates in a 1536-well plate.

(A) Image of maximum fluorescence intensity projection of 5 and 24 h time point in 3D real-time imaging of Aβ_{42} aggregates. 25 µM Aβ_{42} and 25 nM QDAβ were incubated for 24 h. Images were captured using a confocal microscope. Bars = 10 µm. (B) Quantification of fluorescence intensity of Supplementary Movie 7. Fluorescence intensity was measured from 5 h after the morphology of Aβ_{42} did not change. Note that fluorescence intensity increased over time. Error bars represent ±SDs of the mean values of fluorescence intensities of four separated areas in the image.

Supplementary Movie Legends

Supplementary Movie S1. PC12 cells were co-incubated with 20 μM $A\beta_{42}$ and 30 nM QDAβ. This movie shows the aggregation of $A\beta_{42}$ around PC12 cells for 48 h. Still images are displayed in Fig. 2A.

Supplementary Movie S2. This movie shows a 3D reconstruction image of the 48 h time point in Fig. 2A. Note that no $A\beta_{42}$ aggregates exist on top of the cell.

Supplementary Movie S3. PC12 cells were co-incubated with 20 μM $A\beta_{42}$ and 30 nM QDAβ. This movie shows movement of cell protrusion (left) and aggregation of $A\beta_{42}$ (right). Images were captured at a rate of 1 frame/10 min. Still images are displayed in Fig. 3A.

Supplementary Movie S4. PC12 cells were co-incubated with 16 μM $A\beta_{42}$ and 30 nM QDAβ. Maximum fluorescence intensity projection movie shows aggregation of $A\beta_{42}$ around the PC12 cell. Images were captured at a rate of 1 frame/15 min. Still images are displayed in Fig. 4A.

Supplementary Movie S5. PC12 cells were co-incubated with 16 μM $A\beta_{42}$ and 30 nM QDAβ. 3D reconstruction movie of Movie S4 shows promotion of $A\beta_{42}$ aggregation on the cell surface where an active protrusion formed. Images were captured at a rate of 1 frame/15 min. Still images are displayed in Fig. 4B.

Supplementary Movie S6. Scratched PC12 cells monolayer were incubated with 1% DMSO or 25 μM $A\beta_{42}$ and 30 nM QDAβ. WHA movies show migrating PC12 cells for 24 h. The promotion of $A\beta_{42}$ aggregation was occurred on the edge of wound. Images were captured at a rate of 1 frame/10 min. Still images are displayed in Fig. 5C and Supplementary Fig. S4.

Supplementary Movie S7. PC12 cells were co-incubated with 16 μM $A\beta_{42}$ and 30 nM QDAβ. 3D reconstruction movie shows death of an $A\beta_{42}$-aggregated cell. Images were captured at a rate of 1 frame/15 min. Still images are displayed in Fig. 6B.

Supplementary Movie S8. This movie shows maximum fluorescence intensity projection of 3D real time imaging of $A\beta_{42}$ aggregation in a 1536-well plate. 25 μM $A\beta_{42}$ and 25 nM QDAβ were incubated for 24 h. Images were captured at a rate of 1 frame/3

min. Still images are displayed in Supplementary Fig. S6A. Bar = 10 μm.
